# How dental students’ course experiences and satisfaction of their basic psychological needs influence passion for studying in Chile

**DOI:** 10.3352/jeehp.2019.16.37

**Published:** 2019-11-29

**Authors:** Cesar Orsini, Jorge Tricio, Doris Tapia, Cristina Segura

**Affiliations:** 1 Faculty Development Office, Faculty of Dentistry, Universidad de los Andes, Las Condes, Chile; 2 Department of Dentistry, Faculty of Medicine and Dentistry, Universidad de Antofagasta, Antofagasta, Chile; 3 Institute of Odontostomatology, Dental School, Faculty of Medicine, Universidad Austral de Chile, Valdivia, Chile; Hallym University, Korea

**Keywords:** Chile, Dental students, Emotions, Frustration, Personal satisfaction

## Abstract

**Purpose:**

This study aimed to determine how the general course experiences of dental students in Chile and the satisfaction or frustration of their basic psychological needs influenced their passion for studying, and how passion influenced students’ study strategies.

**Methods:**

A correlational cross-sectional study was conducted at 3 Chilean dental schools between April and June 2018, in which 935 undergraduate students participated. Students responded to Spanish-language versions of 4 psychological scale tools: the Course Experience Questionnaire, the Basic Psychological Needs Satisfac¬tion and Frustration Scale, the Passion Scale, and the Revised Study Process Questionnaire. Data were analysed with bivariate correlations and structural equation modelling, controlling for age, gender, year of study, and type of university.

**Results:**

Students’ general course experiences (i.e., good teaching, clear goals and standards, appropriate assessment, and appropriate workload) positively predicted basic need satisfaction and negatively predicted need frustration. Need satisfaction positively predicted passion in students, with stronger scores for harmonious passion. Basic need frustration positively predicted obsessive passion and negatively predicted harmonious passion. Harmonious passion positively predicted deep study strategies and negatively predicted surface study strategies, while obsessive passion positively predicted both deep and surface study strategies.

**Conclusion:**

Dental students’ optimal course experiences positively influenced the satisfaction of their basic psychological needs, which favoured harmonious over obsessive passion. In turn, harmonious over obsessive passion positively influenced deep study strategies. Therefore, efforts should be made to provide course experiences that support students’ basic needs and harmonious passion for studying, both in classroom and chair-side teaching.

## Introduction

### Background

Students in the health professions have been characterised as high achievers who are autonomously motivated and devote long hours to their studies [1]. Dental education is no exception to this, as students generally thrive despite a highly demanding, controlling, and stressful academic programme [2]. While some research has been carried out on dental students’ motivation, few empirical investigations have explored the development and consequences of students’ passion for studying [3,4].

According to the dualistic model of passion (DMP), we can define passion as a strong inclination toward a self-defining activity that one loves, finds important and meaningful, and invests time and energy into [5]. The DMP, however, distinguishes between 2 types of passions depending on how students internalise the reasons to engage in an activity [6]. On one end there is obsessive passion, in which someone engages in a passionate activity mainly due to certain contingencies or external reasons (e.g., pressure, ego-involvement, social acceptance, self-esteem) that lead to uncontrollable over-engagement and rigid persistence. Despite leading to some benefits in the long-term, such as improved performance, this rigid persistence may result in conflicts with other aspects of students’ lives and other negative consequences during or after the activity. For instance, a dental student obsessively passionate about studying endodontics may refuse to take part in leisure, sport, or family activities because of the urge to study. Alternatively, when finally engaging in other activities, the student may be unable to stop feeling guilty or distracted because he or she is not studying. On the other end, there is harmonious passion, in which someone engages in a passionate activity, mainly due to personal endorsement, choice, volition, and valuing the activity. This passion is present even without any associated external pressures, thus leading to flexible engagement and positive consequences during and after the activity (e.g., concentration, flow, positive affect, satisfaction). For instance, a dental student harmoniously passionate about studying oral surgery might plan his or her working hours to leave time for other activities, thus resulting in full immersion while studying and while taking part in other activities.

Being passionate is essential for academic success; however, the interactions and inputs received from the educational environment may facilitate harmonious or obsessive passion [7]. In line with self-determination theory, an educational environment that fosters students’ basic psychological needs of autonomy (engaging out of choice and valuing activities), competence (feeling capable), and relatedness (feeling cared for and significant in one’s environment) will facilitate the autonomous internalisation of reasons to engage in activities, favouring harmonious over obsessive passion. On the contrary, if these basic needs are frustrated, the reasons to engage in activities will be partly internalised and controlled, facilitating obsessive rather than harmonious passion [8]. In this sense, a learning environment that promotes need satisfaction and harmonious passion might positively influence students’ learning and favour a deep over a surface study strategy. A deep study strategy refers to an approach to understanding and maximising meaning, while a surface study strategy refers to students favouring memorisation and rote learning [9].

### Purpose

**Fig. 1.**
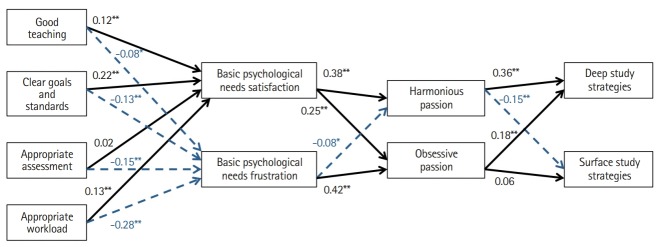
Hypothesised model depicting the expected associations between students’ course experiences, their basic psychological needs, passion towards studying, and study strategies. Black bolded arrows represent hypothesised positive associations, whereas blue dotted arrows represent hypothesised negative associations.

This study sought to examine how dental students’ course experiences and the satisfaction or frustration of their basic psychological needs influenced passion for studying, and how passion influenced students’ study strategies. We tested the following hypotheses presented in the model in Fig. 1, which was developed based on the theoretical framework of DMP and self-determination theory: (1) Students’ positive perceptions of course experiences (i.e., good teaching, clear goals and standards, appropriate assessment, and appropriate workload) positively predict basic need satisfaction; (2) Satisfaction of students’ basic psychological needs positively predicts harmonious passion; and (3) Students’ harmonious passion positively predicts deep study strategies.

## Methods

### Ethics statement

The ethics committee of the Universidad de Los Andes approved this study (reference: CECFM201508).

### Study design

This is a correlational cross-sectional study.

### Participants and procedure

All undergraduate dental students (BDS) from year 1 to 6 at the Universidad de Los Andes, Universidad Austral, and Universidad de Antofagasta in Chile were invited to participate between April and June 2018 (n=1,252). These institutions are all members of the Association for Dental Education in Chile and therefore deliver similar discipline-based curricula comprising 2 years of basic sciences, 1 year of preclinical studies, and 3 years of clinical sciences and practice. At each dental school, students answered a paper-and-pencil questionnaire containing 4 psychological scales.

### Sample size

A power analysis was conducted to be able to identify small effects and to reduce the odds of type II error. According to this, our minimum final sample was determined to be 759 students for correlation and structural equation modelling (SEM) analyses (α=0.05, power=0.80; G*Power software ver. 3.1.9.2; Heinrich-Heine-Universität Düsseldorf, Düsseldorf, Germany; http://www.gpower.hhu.de/) (Dataset 1).

### Measures

Students were presented with Spanish-language versions of 4 survey tools that contained 78 items in total and were asked to indicate their gender, age, and year of study. Course experiences were measured with the 22-item Spanish Course Experience Questionnaire [10], which included subscales for good teaching, clear goals and standards, appropriate assessment, and appropriate workload (Supplement 1). This questionnaire was adapted to measure students’ general experiences and not a specific course within the dental curriculum. Basic psychological needs were examined using the 24-item Spanish Basic Psychological Needs Satisfac¬tion and Frustration Scale. The basic needs of autonomy, competence, and relatedness were assessed using the subscales of need satisfaction and need frustration [11]. Passion for studying was measured using the 12-item Spanish Passion Scale for Studying, which included subscales for harmonious and obsessive passion [12]. Finally, study strategies were assessed with the 20-item Spanish Revised Study Process Questionnaire, which included subscales for deep and surface study strategies [13]. All instruments were adapted to refer to the dental education context and students’ experiences in their current semester. Responses to the Spanish Passion Scale for Studying were on a 7-point Likert scale, while the other 3 scales were answered on a 5-point Likert scale. A higher score indicated higher endorsement of the corresponding variable (Supplement 1). All instruments have shown acceptable reliability and validity in previous studies using similar samples [10-13] . These were free to use for academic purposes with provision of the corresponding references.

### Statistical analysis

The α level was set at ≤0.05, and all data analyses were conducted using IBM SPSS ver. 22.00 (IBM Corp., Armonk, NY, USA) and AMOS ver. 20.0 (IBM Corp.). Cronbach α scores and the Pearson correlation coefficient were used to calculate internal consistency and bivariate correlations. These were computed for all measures, along with descriptive scores. Afterwards, SEM analysis was used to test the model’s fit to the data and to interpret the hypothesized paths as described by Kline [14] (Fig. 1). As there are no gold-standard scores for a definitive evaluation of SEM results, we conducted the following series of tests (acceptable cut-off score in parentheses): the chi-square test (χ^2^ >0.05), the standardized root mean square residual (SRMR <0.05), the comparative fit index (CFI >0.90), and the goodness-of-fit index (GFI >0.90). Additionally, we compared the goodness of fit of the hypothesised model to that of an alternative model where the order of basic need satisfaction/frustration and harmonious/obsessive passion were interchanged. Standardized path scores of parameter estimates were interpreted as regression coefficients. In addition, mean differences with regard to gender, year of study, and type of university (private or public) were computed through the independent t-test with the Holm-Bonferroni correction since previous research has suggested that they may have confounding effects on students’ learning experiences [1]. These resulted in significant differences (P<0.05) and therefore gender, year of study, and type of university were added as controls to the SEM analysis.

## Results

In total, 935 students agreed to participate (response rate, 74.7%), with an average age of 21.4 years (standard deviation, 2.5 years; range, 17–32 years). Of the respondents, 65% (n=608) were women and 35% (n=327) were men, and 67.1% (n=627) came from private institutions, while 32.9% (n=308) came from a public institution. The distribution of year of study was as follows: first-year, 21.7% (n=203); second-year, 19.4% (n=181); third-year, 16.4% (n=153); fourth-year, 19.3% (n=180); fifth-year, 13.5% (n=126); and sixth-year, 9.8% (n=92). These distributions broadly corresponded to the institutions’ demographics, except for sixth-year students, who were less represented because of their outreach activities. Raw data are available in Dataset 1.

### Internal consistency, means, and bivariate correlations

These results are shown in Table 1. The α coefficients were above the standard for acceptance (0.60), suggesting that the scales were reliable within the context of this study. The 2 exceptions were the subscales of appropriate assessment and appropriate workload, both of which were below, but close to, 0.60. Nonetheless, these were included in the analyses considering their complexity and the fact that they contained few items, which might have inherently worked against their reliability. Students reported positive course experiences, with all scores above the mean of the corresponding subscales, except for appropriate workload. Basic psychological needs were reported as more satisfied than frustrated, and harmonious passion and deep study strategies showed higher scores than obsessive passion and surface study strategies, respectively. With regards to correlations, all 4 components of students’ course experiences showed significant positive correlations with basic need satisfaction and significant negative correlations with need frustration (Table 1). Students’ basic need satisfaction showed a significant positive correlation with harmonious passion (r=0.51, P<0.01) and a negative nonsignificant correlation with obsessive passion (r=-0.03, P>0.05). Basic need frustration, in contrast, showed a significant negative correlation with harmonious passion (r=-0.42, P<0.01) and a significant positive correlation with obsessive passion (r=0.27, P<0.01). Harmonious and obsessive passions showed a positive correlation, as they both represent passion for studying (r=0.14, P<0.05). Finally, harmonious passion showed a significant positive correlation with deep study strategies (r=0.43, P<0.01) and a significant negative correlation with surface study strategies (r=-0.11, P<0.01), while obsessive passion showed a significant positive correlation with deep study strategies (r=0.28, P<0.01) and a significant positive correlation with surface study strategies (r=0.09, P<0.01).

### Structural equation modelling

**Fig. 2.**
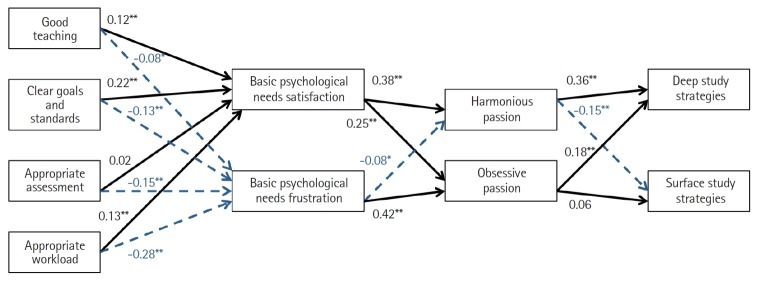
Structural equation model showing standardized regression coefficients in the hypothesized model for all participants. Black bolded arrows represent hypothesised positive associations, whereas blue dotted arrows represent hypothesised negative associations. The residuals, covariances, and regression paths of control variables have been omitted to simplify the model’s visualization. The significance of all paths is based on unstandardized regression coefficients, controlling for gender, age, year of study, and type of institution (public or private). *P<0.05. **P<0.001.

For fit statistics, the chi-square test was significant (χ^2^=206.754, degrees of freedom=16, P<0.001) suggesting a poor fit. This statistic, however, has been reported as sensitive to large samples, where small differences may result in significant scores. The CFI (0.949), GFI (0.971), and SRMR (0.047), which are not influenced by sample size, were all above the standard for acceptance, suggesting an adequate fit of the model to the observed data. In addition, the abovementioned alternative model showed poorer fit indices (CFI, 0.823; GFI, 0.916; SRMR, 0.069). Therefore, the parameter estimates of the hypothesised model were retained. As shown in Fig. 2, all standardized regression paths were in the hypothesized direction, controlling for the effects of age, gender, year of study, and type of university. Good teaching, clear goals and standards, appropriate assessment, and appropriate workload were all positive and significant predictors of the satisfaction of students’ basic psychological needs and significant negative predictors of their frustration. The single exception was the association between appropriate assessment and basic need satisfaction, which was positive but nonsignificant. Basic need satisfaction was a significant positive predictor of both types of passion. However, the association was stronger with harmonious than with obsessive passion. Basic need frustration, in contrast, was a significant positive predictor of obsessive passion and a significant negative predictor of harmonious passion. Finally, both types of passion were significant positive predictors of students’ deep study strategies. However, harmonious passion was a stronger predictor than obsessive passion. With regards to surface study strategies, harmonious passion was a significant negative predictor, while obsessive passion showed a positive but nonsignificant association.

## Discussion

### Interpretation and suggestions

According to the above results, hypothesis 1 was accepted. An optimal course experience was positively associated with the satisfaction of students’ basic needs and prevented their frustration. This result coincides with previous research in dental education, in which a learning climate and feedback that supported students’ basic needs was found to predict autonomous motivation [15]. This emphasises the relevance of regularly assessing how students and faculty perceive the learning environment in aspects such as quality of teaching and assessment, workload, and clear and explicit standards, amongst others [16].

Hypothesis 2 was also accepted since students’ perceiving their basic needs as satisfied was positively associated with passion towards studying, with a predominance of harmonious over obsessive passion. Moreover, an important finding was that students perceiving their needs as deliberately or unintentionally frustrated was found to facilitate obsessive passion and prevent harmonious passion. These findings are consistent with research conducted in other educational domains showing that students’ harmonious or obsessive passion is influenced by how they internalise their reasons to engage in activities, and consequently by the satisfaction or frustration of their basic psychological needs [7]. This also underscores the relevance of supporting students’ self-regulation before, during, and after a learning activity or clinical procedure, as a way to promote students’ autonomy and to reflect how and why they are performing it [17].

Finally, concerning hypothesis 3, it was found that both types of passions predicted deep study strategies in students; however, harmonious passion showed a stronger association with deep strategies and was found to prevent a surface study approach. This is relevant, as it agrees with previous research showing that obsessive passion leads to some positive outcomes; however, being harmoniously passionate for studying leads students to more strongly adopt a study approach that seeks meaning and understanding, and prevents the adoption of an approach focused on rote learning and memorising [6,13].

These findings, while preliminary, provide support for the DMP in dental education and suggest that students’ type of passion is important for their professional development. This is especially relevant considering the intensity and long hours that students must devote throughout the rewarding and interesting, but also demanding, dental curriculum [2]. Within this academic programme, students might find themselves passionate and immersed. However, this passion may become obsessive and eventually lead to some positive outcomes, as described above, but at what cost? While both types of passion might lead to some positive behavioural outcomes, the rigid persistence and over-engagement of obsessive passion have been shown to result in a cost in terms of psychological well-being, as shown by lower levels of concentration, satisfaction, emotions, higher burnout, and conflicts with other aspects of one’s life and social interactions [3,7]. On the contrary, harmonious passion has been shown to lead to more positive behavioural outcomes, with the added benefits of happiness and a psychologically sound experience [6].

This raises the question of how teachers and curriculum developers can design environments that contribute to dental students’ flexibility and foster control over their passionate activity and not the other way around. According to the DMP, 3 aspects are critical for facilitating harmonious over obsessive passion. The first aspect is to support activity valuation by providing a clear rationale for the proposed activities and to leave space for students to find and develop their areas of interest and passion [7]. This not only means that teachers should show passion, be enthusiastic, and explain the relevance of activities to students, but also that they should carefully plan activities and assessments without overloading students. The second aspect is to promote identification with the activity so that students see themselves as part of their community of practice and feel they are “dentists” [6,7]. Early patient contact experiences and situated learning may support this identification. The third aspect is to plan activities and interact with students in a way that supports their basic psychological needs of feeling autonomous, competent, and related to important others. A comprehensive set of strategies on how to teach dental students in a needs-supportive way was presented by Orsini et al. [18]. Consequently, when present, these 3 aspects of the learning environment will facilitate an autonomous internalisation of activities and contribute to harmonious over obsessive passion for studying.

### Limitations

Despite adding to our understanding of students’ passion for studying in the health professions, this study was limited by its exploratory and cross-sectional nature. The majority of the correlation coefficients and regressions paths from SEM corresponded to small and medium-size effects; therefore, future research should incorporate other variables that might contribute to explaining the observed variance, such as affective consequences and intrapersonal factors, and should deploy longitudinal methods in order to build a broader framework on how passion influences students’ learning experiences in clinical settings. This study focused on exploring the associations amongst a set of relevant variables; however, it would be interesting for future research to characterise students’ passions (or lack thereof) according to their demographics and different stages throughout the dental curriculum.

### Conclusion

The study shows that optimal course experiences among students were associated with satisfaction, instead of frustration, of their basic psychological needs of feeling autonomous, competent, and related to important others. Satisfaction of students’ basic needs was found to favour a harmonious over an obsessive passion for studying, which was associated to a deep over a surface study approach. In light of these findings, efforts should be made to create clinical learning environments that support students’ basic needs and harmonious passion, as this may be crucial for understanding why students persist and thrive in academic activities when times are rough or when it is not easy to do.

## Figures and Tables

**Table 1.** Bivariate correlations, internal consistency, and mean values (SD) of all measures

	GT	CGS	AA	AW	NS	NF	HP	OP	DSS	SSS	Age
GT	-	0.49^**^	0.06	0.22^**^	0.25^**^	-0.18^**^	0.41^**^	0.13^**^	0.32^**^	0.01	-0.20^**^
CGS		-	0.13^**^	0.16^**^	0.29^**^	-0.22^**^	0.34^**^	0.07^*^	0.26^**^	-0.10^**^	-0.17^**^
AA			-	0.18^**^	0.07^*^	-0.23^**^	0.04	-0.17^**^	-0.07^*^	-0.26^**^	0.05
AW				-	0.19^**^	-0.34^**^	0.38^**^	-0.21^**^	0.09^**^	-0.08^*^	-0.01
NS					-	-0.67^**^	0.51^**^	-0.03	0.35^**^	-0.16^**^	0.02
NF						-	-0.42^**^	0.27^**^	-0.20^**^	0.31^**^	-0.04
HP							-	0.14^*^	0.43^**^	-0.11^**^	-0.14^**^
OP								-	0.28^**^	0.09^**^	-0.14^**^
DSS									-	-0.15^**^	-0.21^**^
SSS										-	-0.08^*^
Age											-
α	0.806	0.640	0.595	0.595	0.849	0.827	0.827	0.636	0.781	0.775	-
Mean±SD	2.91±0.75	3.51±0.71	2.95±0.88	2.06±0.66	3.93±0.57	2.58±0.67	4.16±1.16	3.10±1.01	31.67±6.53	26.55±6.92	21.44±2.53

GT, good teaching; CGS, clear goals and standards; AA, appropriate assessment; AW, appropriate workload; NS, basic psychological needs satisfaction; NF, basic psychological needs frustration; HP, harmonious passion; OP, obsessive passion; DSS, deep study strategies; SSS, surface study strategies; SD, standard deviation.

* P<0.05 (2-tailed).

** P<0.01 (2-tailed).

## Footnotes

**Data availability** Data files are available from https://doi.org/10.7910/DVN/XD2QMD Dataset 1. Raw data

## Supplementary materials

Data files are available from https://doi.org/10.7910/DVN/XD2QMD

Supplement 1. Spanish versions of the scales that were used.

Supplement 2. Audio recording of the abstract.
