# Supplementary material for: How dental students’ course experiences and satisfaction of their basic psychological needs influence passion for studying in Chile
Source: J Educ Eval Health Prof. 2019 Nov 29;16:37. doi: 10.3352/jeehp.2019.16.37 (PMC6920708; doi:10.3352/jeehp.2019.16.37)
Supplement: Supplementary file 2 — Supplement 1. Spanish versions of the scales that were used. [file jeehp-16-37-suppl.pdf]

Universidad de los Andes
FACULTAD DE ODONTOLOGÍA

Datos Demográficos *[Demographic Data]*

Por favor completa los siguientes datos o marca con una X donde corresponda:
[Please complete the following information or mark with an X where applicable:]

**EDAD** *[Age]*		**SEXO** *[Sex]*	Femenino		
			Masculino		

**CURSO** *[Year of Study]*	1º	2º	3º	4º	5º	6º

ESCALA DE PASIÓN HACIA LOS ESTUDIOS UNIVERSITARIOS *[Passion Scale for Studying]*

(Vallerand et al. 2003, Chamarro et al. 2015)

Teniendo en mente el contexto de tus estudios Universitarios en el último semestre y utilizando la siguiente escala, por favor indica marcando con una **X** tu nivel de acuerdo o desacuerdo con cada ítem.
[Keeping in mind the context of your University studies in the current semester and using the following scale, please indicate by marking with X your level of agreement or disagreement with each item.]

Totalmente en desacuerdo	Muy poco de acuerdo	Un poco de Acuerdo	Moderadamente de acuerdo	Bastante de acuerdo	Muy de acuerdo	Totalmente de Acuerdo
**1**	**2**	**3**	**4**	**5**	**6**	**7**
[Strongly disagree]						*[Strongly agree]*

	**¿Cuán de acuerdo o desacuerdo estás con cada ítem con respecto a tus estudios Universitarios?** *[How much do you agree or disagree with each item regarding your University studies?]*							
1	Mis estudios Universitarios están en armonía con las otras actividades de mi vida. *[My university studies are in harmony with the other activities in my life]*	1	2	3	4	5	6	7
2	Tengo dificultad para controlar la urgencia que siento por mis estudios Universitarios. *[I have difficulties controlling the urge for my university studies]*	1	2	3	4	5	6	7
3	Las cosas nuevas que descubro a través de mis estudios Universitarios me permiten apreciarlos todavía más. *[The new things that I discover in my university studies allow me to appreciate it even more]*	1	2	3	4	5	6	7
4	Tengo casi un sentimiento obsesivo respecto a mis estudios Universitarios. *[I have almost an obsessive feeling for my university studies]*	1	2	3	4	5	6	7
5	Mis estudios Universitarios reflejan las cualidades que me gustan de mí mismo/a. *[My university studies reflect the qualities I like about in myself]*	1	2	3	4	5	6	7
6	Mis estudios Universitarios me permiten vivir una variedad de experiencias. *[My university studies allow me to live a variety of experiences]*	1	2	3	4	5	6	7
7	Mis estudios Universitarios son lo único que verdaderamente me activa. *[My university studies are the only thing that really turns me on]*	1	2	3	4	5	6	7
8	Mis estudios Universitarios están bien integrados en mi vida. *[My university studies are well integrated in my life]*	1	2	3	4	5	6	7
9	Si pudiera, solo estaría dedicado a mis estudios Universitarios. *[If I could, I would only dedicate to my university studies]*	1	2	3	4	5	6	7
10	Mis estudios Universitarios están en armonía con otras cosas que son parte de mí. *[My university studies are in harmony with other things that are part of me]*	1	2	3	4	5	6	7
11	Mis estudios Universitarios son tan excitantes que a veces pierdo el control sobre ellos. *[My university studies are so exciting that I sometimes lose control over them]*	1	2	3	4	5	6	7
12	Tengo la impresión de que mis estudios Universitarios me controlan. *[I have the impression that my university studies control me]*	1	2	3	4	5	6	7

CUESTIONARIO DE LA EXPERIENCIA EN LOS CURSOS (CEQ) *[Course Experience Questionnaire]*
(Ramsden 2006, González et al. 2012)

Por favor responde todas las preguntas marcando con una **X**. No pierdas mucho tiempo en cada pregunta. Tu primera reacción es probablemente la más cercana a la realidad. *[Please answer all questions by marking with an X. Do not waste much time on each question. Your first reaction is probably the closest to reality.]*

	**Con respecto a tu experiencia en los cursos del semestre recién pasado, ¿qué tan cierto es que…?** *[Regarding your experience in the courses of the last semester, how true is that ...]*	Rara vez o nunca *[Rarely or Never]*	A veces *[Sometimes]*	La mitad de las veces *[Half of times]*	Frecuente-mente *[Frequently]*	Casi siempre o siempre *[Almost Always or Always]*
1	Los profesores me motivan a hacer mi mejor esfuerzo. *[Teachers motivate me to do my best.]*	1	2	3	4	5
2	Hay una gran presión sobre mí como estudiante. *[There is great pressure on me as a student.]*	1	2	3	4	5
3	Cuando se usan las TICs (Tecnologías de la Información y la Comunicación, ejemplo MOODLE) me han ayudado a aprender. *[When ICTs are used (Information and Communication Technologies, example MOODLE) they have helped me learn.*	1	2	3	4	5
4	Siempre es fácil conocer las exigencias del trabajo esperado en los cursos. *[It is always easy to know the demands of the expected work in the courses.]*	1	2	3	4	5
5	Desde el inicio de los cursos los profesores dejan en claro qué se esperaba de los estudiantes. *[From the beginning of the courses, teachers make clear what was expected of the students.]*	1	2	3	4	5
6	Los profesores trabajan duro para hacer interesante la materia. *[Teachers work hard to make the subject interesting.]*	1	2	3	4	5
7	Los profesores han sido muy buenos explicando la materia. *[Teachers have been very good at explaining the subject.]*	1	2	3	4	5
8	Mis experiencias en línea (con TICs) me ayudan a involucrarme activamente con mi aprendizaje. *[My online experiences (with ICTs) help me to get actively involved with my learning.]*	1	2	3	4	5
9	Los profesores sólo me hacen preguntas literales de la materia. *[Teachers only ask me literal questions about the subject.]*	1	2	3	4	5
10	Los profesores normalmente me dan retroalimentación útil sobre cómo lo estoy haciendo. *[Teachers usually give me useful feedback on how I am doing it.]*	1	2	3	4	5
11	En los cursos he tenido una idea clara hacia dónde vamos y qué se esperaba de mí. *[In the courses I had a clear idea where we are going and what was expected of me.]*	1	2	3	4	5
12	Para hacerlo bien en los cursos todo lo que necesito es tener buena memoria. *[To do well in the courses all I need is to have a good memory.]*	1	2	3	4	5
13	A menudo es difícil descubrir qué se espera de mí en los cursos. *[It is often difficult to discover what is expected of me in the courses.]*	1	2	3	4	5
14	Las experiencias de aprendizaje en línea (con TICS) de los cursos han estado bien integradas con mi aprendizaje presencial. *[The online learning experiences (with TICS) have been well integrated with my face-to-face learning.]*	1	2	3	4	5
15	La carga de trabajo es muy pesada. *[The workload is very heavy.]*	1	2	3	4	5
16	Los profesores emplean bastante tiempo para comentar mis trabajos. *[Teachers spend a lot of time commenting on my work.]*	1	2	3	4	5
17	Los profesores hacen un esfuerzo real para tratar de entender las dificultades que yo tengo con mi estudio. *[Teachers make a real effort trying to understand the difficulties that I have with my study.]*	1	2	3	4	5
18	Los recursos de la página web de mi universidad (plataforma en línea, biblioteca, sitios web de curso, etc.) apoyan mi aprendizaje. *[The resources of my university's website support my learning.]*	1	2	3	4	5
19	Generalmente me dan el tiempo suficiente para comprender las cosas que tengo que aprender. *[I usually get enough time to understand the things I have to learn.]*	1	2	3	4	5
20	El gran volumen de trabajo contemplado significa que no todo ha podido ser comprendido en detalle. *[The large volume of work contemplated means that not everything has been understood in detail.]*	1	2	3	4	5
21	La comunicación en línea entre estudiantes y profesores ayuda a mi aprendizaje. *[Online communication between students and teachers helps my learning.]*	1	2	3	4	5
22	Los profesores parecen más interesados en evaluar lo que memorizo por sobre lo que he comprendido. *[Teachers seem more interested in evaluating what I memorize over what I have understood.]*	1	2	3	4	5

CUESTIONARIO SOBRE SATISFACCIÓN Y FRUSTRACIÓN DE NECESIDADES PSICOLÓGICAS BÁSICAS EN LA UNIVERSIDAD Basic *[Psychological Need Satisfaction and Frustration Scale at University]*

(Chen et al, 2015)

A continuación, queremos preguntarte **cómo te sientes actualmente en la Universidad**. Por favor, lee cada una de las siguientes afirmaciones cuidadosamente. Puedes marcar con una **X** una respuesta entre el 1 (totalmente falso) y el 5 (totalmente verdadero) para señalar el grado en que cada afirmación es verdadero para ti en este momento de tu vida Universitaria. *[Next, we want to ask you how you currently feel at the University. Please read each of the following statements carefully. You can mark with an X an answer between 1 (totally false) and 5 (totally true) to indicate the degree to which each statement is true for you at this moment in your University life.]*

**1** **Totalmente Falso** *[Totally False]*	**2**	**3**	**4**				**5** **Totalmente Verdadero** *[Totally true]*
1	Siento que tengo libertad y posibilidad de elección en las cosas que hago. *[I feel a sense of choice and freedom in the things I undertake]*		1	2	3	4	5
2	Siento que la mayoría de las cosas que hago, las hago porque "tengo que hacerlas". *[Most of the things I do feel like "I have to"]*		1	2	3	4	5
3	Siento que le importo a las personas que me importan. *[I feel that the people I care about also care about me]*		1	2	3	4	5
4	Me siento excluido del grupo al que quiero pertenecer. *[I feel excluded from the group I want to belong to]*		1	2	3	4	5
5	Siento que soy capaz de hacer las cosas bien. *[I feel confident that I can do things well]*		1	2	3	4	5
6	Tengo serias dudas acerca de si puedo hacer las cosas bien. *[I have serious doubts about whether I can do things well]*		1	2	3	4	5
7	Siento que mis decisiones reflejan lo que realmente quiero. *[I feel that my decisions reflect what I really want]*		1	2	3	4	5
8	Me siento forzado a hacer muchas cosas que yo no elegiría hacer. *[I feel forced to do many things I wouldn't choose to do]*		1	2	3	4	5
9	Me siento conectado con las personas que se preocupan por mí y por las cuales yo me preocupo [I feel connected with people who care for me, and for whom I care]		1	2	3	4	5
10	Siento que las personas que son importantes para mí, son frías y distantes conmigo. *[I feel that people who are important to me are cold and distant towards me]*		1	2	3	4	5
11	Me siento capaz en lo que hago. [I feel capable at what I do]		1	2	3	4	5
12	Me siento decepcionado con gran parte de mi rendimiento. *[I feel disappointed with many of my performance]*		1	2	3	4	5
13	Siento que las cosas que elijo, expresan realmente quién soy. *[I feel my choices express who I really am]*		1	2	3	4	5
14	Me siento presionado a hacer muchas cosas. *[I feel pressured to do too many things]*		1	2	3	4	5
15	Me siento cercano y conectado con otras personas que son importantes para mí. *[I feel close and connected with other people who are important to me]*		1	2	3	4	5
16	Tengo la impresión de que le disgusto a la gente con la que paso tiempo. *[I have the impression that people I spend time with dislike me]*		1	2	3	4	5
17	Me siento competente para alcanzar mis metas. *[I feel competent to achieve my goals]*		1	2	3	4	5
18	Me siento inseguro de mis capacidades. *[I feel insecure about my abilities]*		1	2	3	4	5
19	Siento que he estado haciendo lo que realmente me interesa. *[I feel I have been doing what really interests me]*		1	2	3	4	5
20	Mis actividades diarias se sienten como una cadena de obligaciones. *[My daily activities feel like a chain of obligations]*		1	2	3	4	5
21	Experimento una sensación de calidez cuando estoy con las personas con las que paso tiempo. *[I experience a warm feeling with the people I spend time with]*		1	2	3	4	5
22	Siento que las relaciones interpersonales que tengo son solo superficiales. *[I feel the relationships I have are just superficial]*		1	2	3	4	5
23	Siento que puedo realizar exitosamente tareas difíciles. *[I feel I can successfully complete difficult tasks]*		1	2	3	4	5
24	Me siento como un fracasado por los errores que cometo. *[I feel like a failure because of the mistakes I make]*		1	2	3	4	5

CUESTIONARIO DEL PROCESO DE ESTUDIO REVISADO (R-SPQ-2F)

(Biggs, Kember & Leung, 2001, Justicia et al, 2008)

Señale, con una **X**, la respuesta que mejor refleje su primera reacción. No se preocupe por dar una buena imagen; sus respuestas son confidenciales. *[Indicate, with an X, the answer that best reflects your first reaction. Don't worry about giving a good image; Your answers are confidential.]*

**Con respecto a tu experiencia en los cursos del semestre recién pasado y actual, ¿qué tan cierto es que…?** *[Regarding your experience in the courses of the last semester, how true is it that…?]*		Rara vez o nunca *[Rarely or Never]*	A veces *[Sometimes]*	La mitad de las veces *[Half of times]*	Frecuente-mente *[Frequently]*	Casi siempre o siempre *[Almost Always or Always]*
1	En ocasiones el estudio me proporciona un sentimiento de profunda satisfacción personal. *[I find that at times studying gives me a feeling of deep personal satisfaction]*	1	2	3	4	5
2	Tengo que trabajar bastante en un tema para poder formarme mis propias conclusiones; sólo así me siento satisfecho/a. *[I find that I have to do enough work on a topic so that I can form my own conclusions before I am satisfied]*	1	2	3	4	5
3	Mi objetivo es aprobar los cursos haciendo el menor trabajo posible. *[My aim is to pass the course while doing as little work as possible]*	1	2	3	4	5
4	Sólo estudio en serio lo que se ve en la clase o lo que está en el programa del curso. *[I only study seriously what's given out in class or in the course outlines]*	1	2	3	4	5
5	Creo que cualquier tema de los cursos puede ser interesante una vez que me pongo a trabajar en él. *[I feel that virtually any topic can be highly interesting once I get into it]*	1	2	3	4	5
6	La mayoría de los temas nuevos los encuentro interesantes y frecuentemente paso tiempo extra tratando de obtener más información acerca de ellos. *[I find most new topics interesting and often spend extra time trying to obtain more information about them]*	1	2	3	4	5
7	Cuando no encuentro un curso interesante, le dedico un mínimo de trabajo. *[I do not find my course very interesting so I keep my work to the minimum]*	1	2	3	4	5
8	Aprendo algunas cosas mecánicamente, repasándolas una y otra vez hasta que las sé de memoria, aunque no las comprenda. *[I learn some things by rote, going over and over them until I know them by heart even if I do not understand them]*	1	2	3	4	5
9	A veces siento que estudiar temas académicos puede ser tan emocionante como una buena novela o película. *[I find that studying academic topics can at times be as exciting as a good novel or movie]*	1	2	3	4	5
10	Me autoevalúo en temas importantes hasta que los entiendo por completo. *[I test myself on important topics until I understand them completely]*	1	2	3	4	5
11	Puedo aprobar la mayoría de los exámenes memorizando partes claves de los temas en vez de intentar comprenderlos. *[I find I can get by in most assessments by memorizing key sections rather than trying to understand them]*	1	2	3	4	5
12	Generalmente me limito a estudiar sólo lo que se establece, porque creo que es innecesario hacer cosas extras. *[I generally restrict my study to what is specifically set as I think it is unnecessary to do anything extra]*	1	2	3	4	5
13	Trabajo duro en mis estudios cuando creo que el material es interesante. *[I work hard at my studies because I find the material interesting]*	1	2	3	4	5
14	Dedico gran parte de mi tiempo libre a recopilar más información sobre temas interesantes ya tratados. *[I spend a lot of my free time finding out more about interesting topics which have been discussed in different classes]*	1	2	3	4	5
15	Creo que no es útil estudiar los temas en profundidad. Eso sólo confunde y hace perder el tiempo, cuando lo único que se necesita es familiarizarse con los temas para aprobarlos. *[I find it is not helpful to study topics in depth. It confuses and wastes time, when all you need is a passing acquaintance with topics]*	1	2	3	4	5
16	Me parece que los profesores no deben esperar que los alumnos pasen mucho tiempo estudiando materias que se sabe que no van a entrar en el examen. *[I believe that lecturers shouldn't expect students to spend significant amounts of time studying material everyone knows won't be examined]*	1	2	3	4	5
17	Asisto a la mayoría de las clases con preguntas en mente de las cuales busco respuesta. *[I come to most classes with questions in mind that I want answering]*	1	2	3	4	5
18	Para mí si tiene sentido revisar la mayoría de las lecturas recomendadas para cada clase. *[I make a point of looking at most of the suggested readings that go with the lectures]*	1	2	3	4	5
19	No tiene sentido estudiar el material que probablemente no va a entrar en el examen. *[I see no point in learning material which is not likely to be in the examination]*	1	2	3	4	5
20	Me parece que la mejor forma de aprobar un examen es tratar de memorizar respuestas a preguntas que probablemente entren en él. *[I find the best way to pass*	1	2	3	4	5
